# Building, Hosting and Recruiting: A Brief Introduction to Running Behavioral Experiments Online

**DOI:** 10.3390/brainsci10040251

**Published:** 2020-04-24

**Authors:** Marian Sauter, Dejan Draschkow, Wolfgang Mack

**Affiliations:** 1 Institute for Psychology, Bundeswehr University Munich, 85579 Neubiberg, Germany; wolfgang.mack@unibw.de; 2 Department of Psychiatry, Oxford Centre for Human Brain Activity, Wellcome Centre for Integrative Neuroimaging, University of Oxford, Oxford OX3 9DU, UK; dejan.draschkow@psych.ox.ac.uk

**Keywords:** online experiments, behavioral sciences, online methods, remote testing

## Abstract

Researchers have ample reasons to take their experimental studies out of the lab and into the online wilderness. For some, it is out of necessity, due to an unforeseen laboratory closure or difficulties in recruiting on-site participants. Others want to benefit from the large and diverse online population. However, the transition from in-lab to online data acquisition is not trivial and might seem overwhelming at first. To facilitate this transition, we present an overview of actively maintained solutions for the critical components of successful online data acquisition: creating, hosting and recruiting. Our aim is to provide a brief introductory resource and discuss important considerations for researchers who are taking their first steps towards online experimentation.

## 1. Introduction

**Figure 1:**
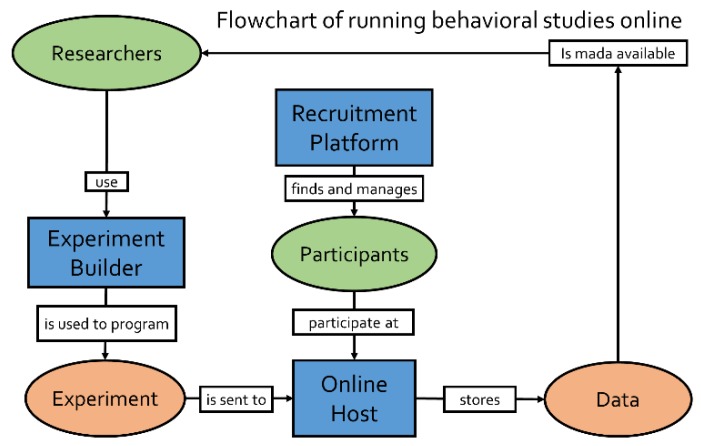
Schematic flow of conducting online experiments. First, experiments are created with an experiment builder. The compiled experimental files are then uploaded to an online host, which generates a link, making the study accessible online (potentially with the aid of a study management system). Participants are recruited through recruitment platforms and access the online experiments on the host. The data is stored on the hosting server.

**Figure 2:**
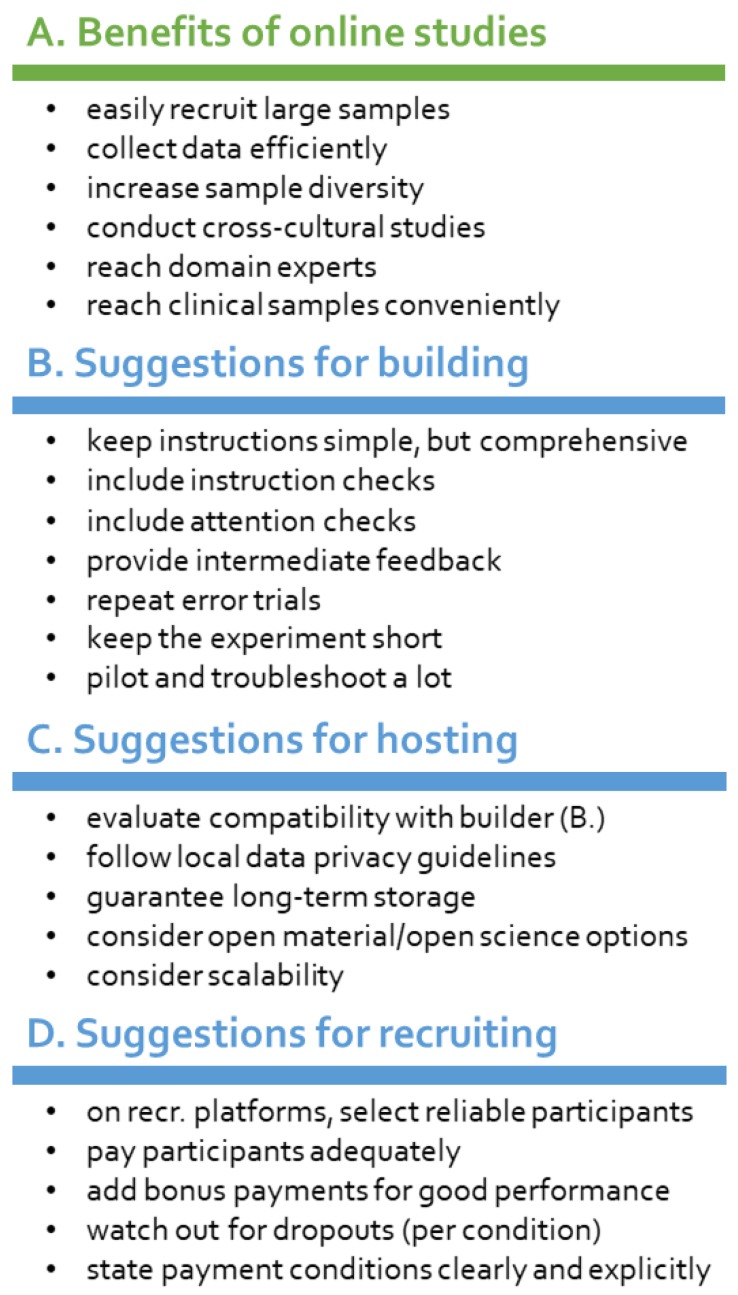
Simplified overview of benefits of online experimentation and recruiting (**A**) as well as suggestions for building online experiments (**B**), hosting online studies (**C**) and recruiting participants online (**D**).

In midst of the Covid-19 pandemic [1], many researchers are bound to rethink lab-based behavioral experiments [2]. There is an emerging need for online testing solutions (for a flowchart, see Figure 1) for day-to-day research activities, thesis work and experimental practical courses alike. Even without a forced shutdown of physical labs, online experiments have gained popularity [3] in the last decade [4,5,6,7,8]. They offer great advantages in terms of participant diversity (in terms of age, gender, origin, culture and social status) [9,10], time and resource efficiency [11]. A big strength of online studies is that they scale very well, as recruiting larger samples does not require a higher workload and particularly hard-to-reach populations become more readily accessible (e.g., [12,13,14,15,16]; see also Figure 2). This can be especially useful for reaching clinical samples or for conducting experimental cross-cultural studies. This article is mainly aimed at cognitive psychology and behavioral neuroscience researchers who have none or limited prior experience in conducting behavioral experiments within an online ecosystem. Our focus is on providing a conceptual overview of the critical components of online experimentation. We further summarize the most well-established tools for implementing these components and provide information about good starting points on the road to online studies. Finally, we offer some considerations and rules of thumb for succeeding with online acquisition, mainly focusing on feasibility and data quality.

## 2. How to Run Behavioral Experiments Online

The critical procedural pillars of any behavioral study are: (1) programming an experiment in the preferred software (e.g., E-prime, PsychoPy, PsychToolbox, etc.); (2) setting-up the testing machine (e.g., lab-computer, multi-unit testing facility, etc.) and (3) recruiting participants to conduct the study. The process of bringing experiments online requires the same pipeline but can be more demanding in terms of harmonizing these steps to ensure that each part of the pipeline is compatible with the other parts (Figure 1). For comprehensibility, we will outline each of these three steps in the next section. This will include a conceptual overview, but also specific examples of solutions (providers, software) which enable the corresponding step in the pipeline. The features and pricing are subject to change. For this reason, in this overview we discuss the main integrative possibilities, which we believe, will not change as quickly (for an up-to-date description of the detailed offerings, one should consult the respective websites). Some of the described solutions are quite modular and specialized (Table 1: B, C, D) in solving only individual steps of the process, whereas other providers offer a more holistic integrated-service ecosystem (Table 1: A). In Section 2.4. we will discuss the considerations one should make when picking an ecosystem, but we will abstain for making strong recommendations and claims at this point. Notably, we limited this overview to software that appears to be under active development to ensure steady security updates (with updates in 2019).

### 2.1. Experiment Builders

Equivalently to studies designed for in-lab testing, the first step in online experimentation is the programming of the experiment (Table 1: B). In comparison to the hegemony of Java, Python, C++ and MATLAB libraries for experimental programming of lab-based studies, Javascript (JS) is the language of choice for online experiments. Even though it is usually ranked as the most popular programming language in the world, JS has not been a hallmark in behavioral testing. Current solutions for online-experimental generation often provide a graphical user interface (GUI), enabling users to drag-and-drop modular components into an experimental sequence. As this rather simplistic, general solution is sometimes insufficiently flexible for more complex experimental designs, a good experimental environment should provide the possibility to extend these modular components with scripts and code-based solutions.

Arguably, the easiest transition from in-lab to online testing is granted by *PsychoPy Builder* [17,18,19,20] and *OpenSesame* [21,22]. Both environments are very popular for traditional testing and allow for a rather straight-forward restructuring towards their online counterparts (*PsychoJS* and *OSWeb*), if only their drag-and-drop modules were used to create experiments. All sections in which scripting was used (e.g., Python inserts) will need to be rewritten into Javascript by the experimenter. Fortunately, Python (especially its ‘object-based’ subset) and Javascript generally only differ in terms of syntax and not programming logic [23], so the rewriting is comparably easy. Additionally, PsychoPy auto-translates base-Python to JS (but not functions from specific libraries). There are plenty other experiment builders available: *Gorilla* [24], *Inquisit Web* [25], *LabVanced* [26] and *Testable* [27], from the integrated-service providers (see Table 1: A) and *lab.js* [28,29], *jsPsych* [30,31], *PsyToolkit* [32,33,34], *tatool web* [35] from the function-specific solutions (see Table 1: B). Their advantages and shortcomings should be evaluated on a lab’s basis depending on individual needs. Generally, as all experiment builders (except for *Inquisit*) operate on a Javascript backend, they offer similar flexibility. They differ in available features (example tasks or modules), but as all builders have online documentations, often with demonstration tasks available, researchers can quickly see whether they fit their specific needs. We see the most difficulties in transferring experiments online for *Psychtoolbox* [36] users, as *MATLAB*^®^’s compile-to-Javascript approach offers no trivial translation of experiments to browser-based software [37].

### 2.2. Hosting and Study Management

In lab-based studies, the final resting place of the finished experiment is the testing machine. For online studies, the experiment needs to be made available for online distribution by hosting it on a server (Table 1: C). This is potentially the most confusing step in the pipeline of creating an online study. Some labs with a lot of experience in online experimentation host their studies on their own servers. This comes with the advantages of low maintenance costs, full control and flexibility. On the downside, it requires some expertise for setup and continued maintenance. The more feasible alternative is centralized hosting providers. Here, hosting and study management is a service, and as such, all providers require a fee. The general idea behind study management systems is to simplify the hosting and participant handling process, like user management, automated data storage or creation of unique participation links.

The whole range of features offered by different providers can be evaluated by visiting their websites. For example, one of the easier but not especially flexible hosting services is offered by *Open Lab* [39]. It takes all studies created with *lab.js* and tests some participants for free. Their unique selling point is arguably its integration with Open Science Framework (OSF) [40]. Participant data are directly uploaded to OSF, which could make it potentially interesting for multi-lab open science initiatives (it should be noted that there is neither a documentation, nor a privacy policy nor information about the responsible person or company publicly available.) Another interesting example is *Pavlovia* [41]. You can upload HTML5/Javascript studies and there is documentation for importing studies created with *lab.js*, *jsPsych* and the *PsychoPy Builder (PsychoJS)*. It offers easy integration with recruitment tools and a GitLab platform [42] where experimenters can share their complete code. An example for more easily setting up one’s own hosting platform is *Just Another Tool for Online Studies (JATOS)* [43,44]. *JATOS* similarly takes HTML5/Javascript studies and documents how to import studies created via *lab.js*, *jsPsych* and *Opensesame Web*. It offers a wide range of options and is a very comprehensive study management tool.

Finally, we want to highlight how important experiment-server compatibility is. In the examples above, we pointed out that a specific hosting service supports studies programmed by specific experimental builders. No host supports all experimental builds and no experimental build is compatible with all hosts. Thus, a decision should always be made on the level of the overall ecosystem and not on the individual components of the pipeline (building vs. hosting vs. recruiting).

### 2.3. Recruitment of Participants

The dominant advantage of running experimental studies online lies in its efficiency. It is feasible to collect responses from hundreds of participants within hours. Thanks to the possibility of world-wide sampling, data collection can literally be completed over night. Once the experiment is created and accessible online (usually with a link), participants can be recruited. Due to higher participant numbers compared to most lab-based studies, handling this process manually is not advisable (for tools see Table 1: D). *ORSEE* [45,46] and *Sona* [47] are participant pool management systems, which offer comprehensive automation tools. However, both require researchers to maintain their own (usually limited in size) participant pool. Additionally, only a limited number of participants can be recruited from the local University, via social media and (institutional) mailing lists. Maintaining an active pool of potential participants is the main advantage of *Amazon Mechanical Turk (MTurk)* [48,49,50], *Prolific Academic* [51,52] and *Qualtrics Panel* [53]. All three providers offer participant recruitment and payment handling services. Note that there is also *CloudResearch* [54], which is a recruitment service that uses the MTurk platform, but unlike MTurk itself, is specifically directed at researchers and offers better participant handling and targeting tools. As one essentially only needs a link to the study, they integrate well with the study management systems and experiment builders mentioned above (see Table 1 for details). While differences in their features are too narrow for the scope of this article, we will discuss some important points on data quality in Section 3.

### 2.4. How to Choose an Ecosystem?

Generally speaking, what researchers need for online experimentation is the same as what they need for lab-based studies (Figure 1): (1) a programmed experiment, (2) a server to host the study and (3) a recruiting platform which advertises to participants. As outlined in the previous sections, there are many solutions for each of these steps. Some solutions provide a single and holistic framework for all three aspects (Table 1: A), whereas other solutions are specifically tailored to one of the aspects and need to be integrated into an ecosystem by the experimenter. Here, the benefits and drawbacks mirror what we already know from software solutions in other domains. Integrated-service providers enable time savings by reducing compatibility issues, providing customer support, and reducing administrative load. On the flip side, they sometimes lack transparency, lack flexibility (minimal compatibility with other solutions), and are generally expensive. Non-profit and open-source solutions usually require more integration considerations and some of them lack direct customer support. Instead, they provide forums and community feedback, low or no costs, and more peer-reviewed benchmarks.

Ideally, the decision on which online ecosystem to use, should be made in accordance to the lab’s capabilities and needs as well as criteria of quality (see Section 3). As all platforms are Javascript-based, they offer similar functionality and most experimental paradigms should theoretically be realizable on all platforms. In principal, a wide range of in-lab research questions can be targeted with online task implementations. The individual journey of a task from in-lab to online, however, can be quite different, as some tasks might need little adjustment, while others would require a major overhaul in order to provide informative results. General recommendations about which tasks are suitable for online testing and which platforms are best for the respective tasks are hard to make, as labs’ use cases are too diverse. It will ultimately be a question of money, the labs’ know-how and specific institutional infrastructure. Of note, switching from other software packages to an integrated-service provider has often the drawback that previously programmed experiments cannot be run anymore and even slight adaptations to the experiments (for example control studies that reviewer 2 asked for) are impossible without completely reprogramming the experiment. Therefore, when deciding how to transfer experiments to the online world, researchers should not only consider what the provider offers, but also how they can adapt their research to the new environment. From an open science perspective, it should also be considered, that not all platforms allow experimental scripts to be exported.

The authors personally had good experiences with *OSWeb* (for building) combined with *JATOS* (for hosting) as well as *PsychoPy* (for building) with *Pavlovia* (for hosting) [55] and *Prolific* (for recruitment). Similarly, the authors would not recommend setting up experimental studies on self-maintained webservers without the aid of a study management system (e.g., *JATOS*) because of the need to account for everything that can go wrong, such as handling data storage, assigning participant codes, assuring participants do not participate more than once, handling payment and so on.

## 3. Data Quality Concerns

The dominant concern with running experiments online is data quality. While the most obvious concerns (e.g., motivation, distractions, stimulus timing) can be dealt with an appropriate design and incentive strategy, we would like to stress the importance of recording and analyzing dropouts [16]. Unlike laboratory studies, participants may drop out at rates of up to 69%. In a dropout analysis of 88 local studies, Zhou and Fishbach [56] found that 20% had a dropout rate of over 30%. Alarmingly, the authors of the analyzed studies were unaware of these dropouts. They also found that out of 289 published MTurk studies, only six disclosed dropout rates. Crucially, dropout rates can interact with the experimental condition [50,56]. To arrive at sound conclusions, it is therefore obligatory to report and analyze dropout rates.

Further, it is imaginable that stimulus presentation times or response times are unreliable because of variations in internet speed or display settings throughout the experiment. However, almost all online solutions operate by downloading (pre-buffering) the entire experiment onto the participant’s machine. Additionally, modern screen refresh rates are almost exclusively set to 60 Hz (de facto standard), making certain specifications of online studies a bit more predictable. Among others [57,58,59], two recent large studies [60,61] investigated timing precision (unintended variability in stimulus presentation) of several online and offline solutions. The online-based comparison found good overall precision for *Gorilla* (13 ms), *jsPsych* (26 ms), *PsychoJS* (−6 ms) and *lab.js* (10 ms). Notably, these means are inflated by particularly bad performance using the Safari browser and Mac OS X. The offline-based comparison, *PsychoPy* and *Opensesame* achieved precisions of 1 ms to 4 ms, with only minor exceptions [60,61], most notably with audio playback.

A study investigating response timing, for example, found an additive timing offset of 87 ms (similar across conditions) in online recordings compared to lab studies, while reproducing all expected task-based effects in various tasks (stroop, flanker, visual search, attentional blink) [62]. In addition to timing, there could be concerns that participants might be less committed when they sit at home and are not directly observed by the experimenter. Several studies have shown that decreased attention to the task is not necessarily found [63,64] and data quality is comparable to lab-based studies [65,66,67,68,69,70,71,72,73]. For example, in a recent study, participants completed several attention checks (in between outcome measures) and there was no difference between lab participants and online participants in any of the measures. However, the study showed that online participants had higher self-reported distraction (use of cell phone, talking to another person, etc). In any case, experimenters should adjust their experiment to account for the sample diversity (see the following section) and participants’ motivations [74]. Crump et al. [50] recommend that the latter can be accomplished by giving accuracy as feedback following each trial; giving prompts to encourage speeded responding when participants do not meet deadlines; and by giving summary assessments of performance after blocks of trials.

## 4. Considerations for Successful Online Studies

There are some aspects researchers should consider when starting out with running online studies or transferring lab-based experiments to online systems [59,75,76] (see Figure 2). To a certain extent, creating successful online experiments is similar to app development: one needs to think of a coherent framework and constantly worry about what the users are doing with the ‘product’ and whether they are using it as intended—without many opportunities for direct feedback. Experimental instructions should be easy enough to be understood by a more diverse sample that is not necessarily used to behavioral testing. Further, measures need to be taken to detect and discourage poor performance, that is ‘fake’ participation. Finally, online studies need to be shorter than classical lab-based studies.

Lab-based studies typically attract young psychology students who are WEIRD (western, educated, and from industrialized, rich, and democratic countries) [77]. The samples drawn from online recruitment platforms are more representative of the general (online) population [9,10]. Study participants have potentially never participated in a behavioral response time experiment. For this reason, experimenters need to be more thorough when creating experimental instructions and ascertain that they can stand on their own without verbal explanations (note: this is also a good recommendation for lab-based studies). It is crucial that the instructions are comprehensible by people of a wider age range representing many cultures and socio-economic backgrounds [10]. In the authors’ experience, a pictorial step-by-step instruction leads to less misunderstandings or even dropouts compared to a single page of text. It is advisable that instructions are forced to stay on the screen for some time before continuation is allowed or an instruction check is added ([50], Experiment 10). In order to check whether participants have truly understood the instructions, a test run and online evaluation before beginning the main experiment is advised. Additionally, study management systems also incorporate some monitoring functions to check that participants stayed on track. For example, it is possible to monitor how often the browser tab running the experiment was minimized during the experiment and viewing distance can be controlled [78]. Notably, on some platforms, explicit measures need to be taken to prevent participants from completing a study twice [79].

The interaction between experimenter and participant is comparably indirect in online experiments. Therefore, participants might be less inclined to be attentive simply for the sake of helping the experimenter with their research. It should therefore be considered to state the relevance of the research explicitly. It was shown that *MTurk* participants perform better, when the task is presented as meaningful [80]. For many participants drawn from recruitment services, the dominant motivation for participation is monetary compensation. While the amount of payment should be similar to lab-based studies for ethical reasons, the data quality is not necessarily affected by higher monetary incentives. In a category learning experiment by Crump et al. [50] (see Experiment 9), participants were paid either $0.75 (low incentive group) or a base amount of $2 and a bonus of up to $2.50 depending on their performance (high incentive group). They found that the incentive structure had no effect on learning or error rates. However, they found that they could collect data more quickly and had fewer dropouts when payment was higher. Typically, participants are paid a fixed amount after successful completion of the study—regardless of how long it takes them to complete it. This is why some participants try to complete the experiment as fast as possible without sticking to the instructions (‘fake’ participation). In order to ensure good data quality, the experimenters might need to adapt the experimental design to discourage such behavior. This implies that the best experiments to run online include a validation mechanism. Generally, forced-choice paradigms (both RT and accuracy types), in which one of the alternatives is the correct choice, are especially suitable because the experimenter can evaluate the participants’ performance during runtime, while judgment studies (e.g., moral dilemma tasks) are harder to evaluate and objective performance or attention checks might need to be included into the design. In the authors experience, an easy option for alternative-forced-choice tasks is to repeat the trial each time participants answered incorrectly. The authors also experienced less dropouts when a progress bar (comparable to surveys) was added. Gamification of the study in general promises to yield better results [81].

Finally, online experimental studies should be short. Participants would possibly not sit 60 min in front of their screen and produce quality data. Since structured investigations are still missing, we asked 103 Germans through *appinio* [82] at which time they would abort an online experiment that offered minimum wage. Most respondents said ‘after 15 minutes’ (44%), followed by ‘after 30 minutes’ (35%), ‘after 45 minutes’ (10%) and ‘after 60 min or never’ (12%).

Keeping these considerations in mind, for a certain subset of investigations (certainly not all), carefully developed online studies have a huge potential. Many of the noise factors can be combated with a large sample size and intelligent preparatory work. Taking behavioral experiments online is facilitated by numerous steadily maintained tools ranging from simple libraries to complex ecosystems. Researchers need to wisely choose the software based on their own prior experience, the lab’s resources and the requirements of the general area of study.

## Figures and Tables

**Table 1:** Simplified comparison of actively maintained tools for online studies in respect to their features, guided integrations (i.e., documentation on website), backends and costs.

	Features and Guided Integrations for			Platform Cost Per		Backend
	Building	Hosting	Recruiting	Month	Participant	
**A. Integrated-Service Providers**						
**Gorilla.sc**	✓	✓, jsP	MTurk, Prolific, SONA, *any*	-	~$1	visual
**Inquisit Web**	✓	✓	✓	~$200	-	visual
**Labvanced**	✓	✓	✓	~ $387	~$1.5	visual
**testable**	✓	✓	✓	n.a. [5]	n.a. [5]	visual
**B. Experiment Builders**						
**jsPsych (jsP)**	✓	JATOS [1], Pavlovia	MTurk	free	free	JS
**lab.js**	✓	JATOS [1], Open Lab, Pavlovia, Qualtrics	MTurk	free	free	visual/JS
**OpenSesame (OS)Web**	✓	JATOS [1]	-	free	free	visual/JS
**PsychoPy Builder (PPB)**	✓	Pavlovia [2]	-	free	free	visual/JS
**PsyToolkit (PsyT)**	✓	✓	SONA, MTurk	free	free	visual/JS
**tatool Web**	✓	✓	MTurk	free	free	visual/JS
**C. Hosts and Study Management**						
**JATOS**	lab.js, jsP, PsyT, OSWeb	(✓) [1]	MTurk, Prolific, [3]	free [1]	free	website
**Pavlovia**	lab.js, jsP, PPB	✓	SONA, Prolific, [3]	~$145	~$0.30	website
**Open Lab**	lab.js	✓	*any* [3]	~$17	free [6]	website
***(inst.) webserver***	lab.js, jsP, OSWeb, PPB	JATOS or none	*any* [3]	free	free	-
**D. Recruitment Services**						
**Amazon MTurk**	*any*	-	✓	-	40%	website
**ORSEE**	*any*	-	(✓) [4]	free	free	website
**SONA**	*any*	-	(✓) [4]	-	n.a. [7]	website
**Prolific Academic**	*any*	-	✓	-	33%	website
**Qualtrics Panel**	Qualtrics/any	-	✓	-	n.a. [7]	website

Note. JS: Javascript; [1] JATOS requires to be installed on your own (institutional) server machine; [2] PsychoPy Builder offers streamlined synchronization with Pavlovia; [3] Links can be shared to any platform or social media but extensive documentation is not available; [4] no active participant pool; [5] testable offers a mixed payment model; testable is also free for all departments in 2020 [38]; [6] up to 300 participants and one study; [7] only available upon request.
